# Multi-Targets: An Unconventional Drug Development Strategy for Alzheimer’s Disease

**DOI:** 10.3389/fnagi.2022.837649

**Published:** 2022-02-09

**Authors:** Cheng-Xin Gong, Chun-Ling Dai, Fei Liu, Khalid Iqbal

**Affiliations:** Department of Neurochemistry, Inge Grundke-Iqbal Research Floor, New York State Institute for Basic Research in Developmental Disabilities, New York, NY, United States

**Keywords:** Alzheimer’s disease, combination therapy, multifactorial hypothesis, multitarget therapy, patient stratification, precision medicine

## Abstract

Alzheimer’s disease (AD) is a progressive neurodegenerative disorder that eventually leads to dementia and death of the patient. Despite the enormous amounts of resources and efforts for AD drug development during the last three decades, no effective treatments have been developed that can slow or halt the progression of the disease. Currently available drugs for treating AD can only improve clinical symptoms temporarily with moderate efficacies. In recent years, the scientific community has realized these challenges and reconsidered the future directions of AD drug development. The most significant recent changes in AD drug development strategy include shifting from amyloid-based targets to other targets, such as tau, and efforts toward better designs for clinical trials. However, most AD drug development is still focused on a single mechanism or target, which is the conventional strategy for drug development. Although multifactorial mechanisms and, on this basis, multi-target strategies have been proposed in recent years, this approach has not been widely recognized and accepted by the mainstream of AD drug development. Here, we emphasize the multifactorial mechanisms of AD and discuss the urgent need for a paradigm shift in AD drug development from a single target to multiple targets, either with the multi-target–directed ligands approach or the combination therapy approach. We hope this article will increase the recognition of the multifactorial nature of AD and promote this paradigm shift. We believe that such a shift will facilitate successful development of effective AD therapies.

## Introduction

Alzheimer’s disease (AD) is a devastating, progressive neurodegenerative disease that causes impaired cognitive function, often combined with psychiatric symptoms such as personality changes, and eventually leads to dementia and death of the patient. Histopathologically, AD is characterized by the accumulation of extracellular deposits of aggregated amyloid β peptides as amyloid plaques and of intracellular neurofibrillary tangles (NFTs) composed of aggregated hyperphosphorylated microtubule-associated protein tau, as well as neuronal and synaptic loss. Only less than 5% of AD cases are the familial form of the disease, caused by mutations in certain genes, such as those for presenilin or amyloid β precursor protein (APP). Most AD cases are sporadic, the etiologies and mechanisms of which are still not known. AD is the sixth leading cause of death (Kochanek et al., 2020) and exerts a huge psychological, social, and economic burden on modern society: the cost for treating and caring for patients with AD and other dementias in 2021 may reach $355 billion in the United States alone (Alzheimer’s Association, 2021).

Despite the extensive resources and efforts invested in AD research and drug development during the last three decades, no effective treatments have been developed that can slow or halt the progression of AD. The currently available drugs for the treatment of AD—cholinesterase inhibitors and memantine—can only improve clinical symptoms temporarily and with moderate efficacy. The clinical efficacy of Aducanumab (Aduhelm™), a passive immunotherapy that received conditional approval from the U.S. Food and Drug Administration (FDA) in June 2021, is very questionable. The demand for new therapeutics that can slow or halt the progression of AD is both great and urgent.

Modern AD research at the molecular level began in the 1970s. The bulk isolation and decoding of the protein composition of NFTs from AD brain was achieved in 1974 (Iqbal et al., 1974), and the central cholinergic deficit in AD was discovered in 1976 (Davies and Maloney, 1976). The discovery of amyloid β peptides as the major components of amyloid plaques, the cloning of APP gene in chromosome 21, and the identification of its mutations in hereditary amyloidosis in 1980s led to the formulation of the amyloid cascade hypothesis (Hardy and Higgins, 1992). Since then, this hypothesis has informed most AD research and drug development. The failure to develop effective drugs that can slow or halt the progression of AD has led to reevaluation of the strategies for AD research and drug development. Many investigators started several years ago to question whether support of the amyloid cascade hypothesis has caused this failure and have shifted their research targets to non-amyloid targets, such as tau, neuroinflammation, and neuroprotection (Iqbal et al., 2016; Cummings et al., 2020). The newly FDA-approved aducanumab (Aduhelm™) that can reduce amyloid β plaques from the brain in mild cognitive impairment (MCI) and mild AD cases but does not show any convincing clinical beneficial effect (US Food and Drug Administration, 2021) further supports this line of thinking. Others blame the failure to develop effective AD drugs on inadequate designs of clinical trials. However, there are currently no unequivocally positive results of any new AD clinical trials targeting molecules other than amyloid, including tau immunotherapy, and with better designs. Almost all of these trials are targeted to a single molecule or mechanism that is potentially or likely involved in the pathogenesis of AD.

More than 95% of all AD cases are sporadic. Sufficient evidence has indicated that sporadic AD has multiple etiologies and involves multiple disease mechanisms (Iqbal et al., 2005). This evidence led us to propose a multifactorial hypothesis for AD, which emphasizes that (i) different causes and mechanisms underlie different populations of AD cases, and (ii) more than one distinct cause and mechanism is involved in individual sporadic AD case (Gong et al., 2018). In light of this multifactorial nature of sporadic AD, it is not surprising that any clinical trials that target a single mechanism would fail, given that would only address a small portion of the study cohort or of the disease mechanisms of an individual case. Therefore, we believe that a paradigm shift of AD drug development from the single-target strategy to a multi-target strategy can dramatically increase the chance of developing effective AD drugs. Here, we discuss the multifactorial nature of AD and argue for the urgent need to shift AD drug development from the conventional single-target strategy to the emerging multi-target strategy.

## Multifactorial Nature of Alzheimer’s Disease

Except for inherent familial AD that accounts for only <5% of the cases, AD is mostly sporadic in nature. There is no single cause or etiology fully responsible for the sporadic form of the disease. Modern research on the etiologies and mechanisms of AD has suggested many players. Therefore, several hypotheses have been proposed, including the amyloid cascade hypothesis, cholinergic hypothesis, tau hypothesis, mitochondrial hypothesis, oxidative stress hypothesis, neuroinflammation hypothesis, brain insulin resistance hypothesis, brain metabolism hypothesis, calcium hypothesis, innate immunity hypothesis, and others (Table 1). Almost all these hypotheses are supported by experimental evidence, but they often dismiss the importance of other alternative factors. The previously proposed hypotheses are likely to be true for only a small population of AD cases (or a specific subgroup of AD cases) or to play only partial role in the pathogenesis of an individual AD case.

**TABLE 1:** Examples of hypotheses proposed for AD.

AD hypothesis	References
Amyloid cascade hypothesis	Hardy and Higgins, 1992, Selkoe and Hardy, 2016
Tau hypothesis	Iqbal and Grundke-Iqbal, 1996, Iqbal et al., 2016
Cholinergic hypothesis	Perry et al., 1977, Bartus et al., 1982
Mitochondrial hypothesis	Moreira et al., 2010, Swerdlow et al., 2010
Oxidative stress hypothesis	Coyle and Puttfarcken, 1993, Zhu et al., 2004
Neuroinflammation hypothesis	McGeer et al., 1994
Brain insulin resistance hypothesis	de la Monte, 2009, Deng et al., 2009
Metabolic hypothesis	Hoyer, 2000, Iqbal and Grundke-Iqbal, 2005, Gong et al., 2016
Calcium hypothesis	Khachaturian, 1994
Innate immunity hypothesis	Guillot-Sestier et al., 2015

**FIGURE 1:**
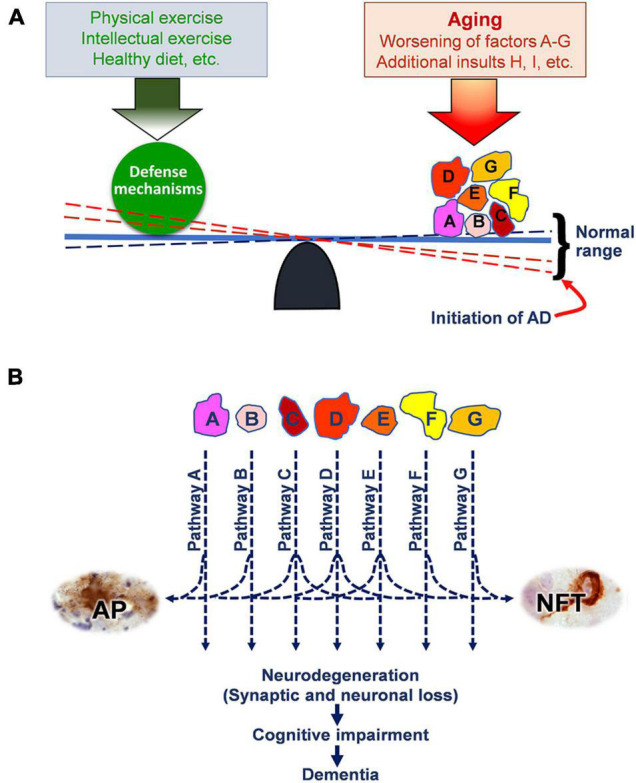
The multifactorial hypothesis of AD. **(A)** The balance between the potential factors/insults accumulated during normal aging and the defense mechanisms. Worsening of these factors/insults (such as A to G) and/or adding of additional insults (such as H, I, etc.) can initiate the onset of AD. Factors/insults A to G represent genetic risk factors and those insults that accumulate during aging, such as mutations in presenilin and APP genes, ApoE4, amyloid β accumulation, oxidative stress, neuroinflammation, etc. Factors/insults H and I represent additional pathological insults, such as brain insulin resistance, repeated traumatic brain injury, and environmental insults. **(B)** The multifactorial insults collectively cause neurodegeneration through multiple molecular mechanisms/pathways and consequently cognitive impairment and dementia. Some of these pathways also lead to the formation of amyloid plaques (APs) and neurofibrillary tangles (NFTs), which are part of the end products of these pathways and are also hallmark brain lesions of AD. Reproduced with permission from Gong et al. (2018), which is available at IOS Press through http://dx.doi.org/10.3233/JAD-179921. The reproduction permission was obtained from IOS Press.

In recognizing the multifactorial nature of AD, we proposed a multifactorial hypothesis for AD in 2018 (Gong et al., 2018). We believe that normal aging is a constant balancing between physiological aging plus pathological risks/insults and the natural defense mechanisms of our body (Figure 1A). Many risks and insults occur and accumulate during aging, including genetic risks, epigenetic and metabolic factors, and environmental insults. The human body also responds to these factors/insults with its defense mechanisms, which could include general defense and those specific to individual insults. The balance between aging/insults and the defense mechanisms is dynamic and can shift within a certain range under physiological conditions. During normal aging, although the right side of the balance shown in Figure 1A can be heavier with the accumulation of factors/insults, such as factors A to G, the balance tilts to the right side but remains within the normal range. However, as one or more of these factors/insults get heavier or new factors/insults (e.g., factor H, I, etc.) are added to them, the imbalance eventually reaches the threshold and breaks the balance, i.e., initiation of the development of AD. These factors/insults collectively result in neurodegeneration, leading to cognitive impairment and eventually dementia, through different molecular pathways (Figure 1B). Some of these pathways are involved in amyloid β overproduction/aggregation and tau hyperphosphorylation/aggregation, leading to the formation of amyloid plaques and NFTs as the two hallmark brain lesions of AD.

This multifactorial hypothesis can nicely explain why aging is the most important risk factor for AD, since the defense mechanisms on the left side of the balance shown in Figure 1A become increasingly weak during aging. On the other hand, healthy lifestyle, such as physical and intellectual exercises and healthy diet, can strengthen the defense mechanisms and thus inhibit or delay the onset of the disease.

It must be emphasized that the development and onset of sporadic AD results from the collective effects of multiple factors/insults—not from one specific insult. This emphasis warrants targeting more than one insult/pathway simultaneously in the development of effective AD treatments. This is probably why none of the previous AD drugs developed on the basis of one single hypothesis/mechanism has been successful. It should also be emphasized that each AD case may have a different combination of etiological factors/insults that cause the onset of AD in this individual. This emphasis recognizes the diversity of etiological factors and molecular mechanisms among individual AD cases, justifies the stratification of AD patients, and explains the presence of significant variations in brain pathologies and in clinical symptoms among various AD cases.

## Multi-Targets: An Unconventional Strategy for Alzheimer’s Disease Drug Development

The single-target approach is preferred or ideal for diseases with single etiological causes or with a clear dominant disease mechanism, such as targeting the pathogens for infectious diseases and hormone replacement treatment for a specific hormone deficiency. For complex disorders, such as AD, intervention at more than one factor/pathway is clearly needed. This approach has been well demonstrated in cancer therapy, in which a combination of several mechanisms has improved clinical outcomes remarkably (Xu et al., 2018). Therefore, such an approach is clearly warranted for AD, a disease of multiple etiological factors and pathogenic mechanisms.

The multi-target strategy for AD drug development has been considered in the last few years but has not yet been widely recognized or implemented by the mainstream. One reason for this might be that the single-target approach is the standard, conventional approach for traditional drug development that is more acceptable by the FDA approval process. Another reason may lie in the challenges in the development of this strategy and in the complexity of evaluating the mechanisms of the drug actions. However, the multifactorial nature of AD demands us to rise to these challenges and to use an unconventional strategy to succeed in AD drug development. There are several approaches for implementing the multi-target strategy for combating AD.

First, design one drug that has two or more functionally active groups or targets. Many natural and synthetic compounds actually have more than one functional group and can target several molecular pathways. For traditional drug development, any activities other than the so-called desired target activity are regarded as non-specific activities. These non-specific activities could actually be beneficial as a drug candidate for AD if they happen to target a molecular pathway that is also involved in the pathogenesis of AD. The specificity of a drug candidate is often hoped to be as high as possible. However, less specific drug candidates might be preferred over the more specific ones for treating multifactorial AD if the “non-specific actions” target other mechanisms involved in AD. For example, glycogen synthase kinase-3β (GSK-3β), the major protein kinase catalyzing tau phosphorylation and probably involved in abnormal hyperphosphorylation of tau, appears to underlie neurodegeneration in AD (Sayas and Avila, 2021). Thus, GSK-3β inhibitors have been investigated and subjected to clinical trials for treating AD (Lovestone et al., 2015). The failure of these trials to show significant clinical benefits might be because this kinase can be replaced by several other tau kinases: more than a dozen protein kinases have been shown to be able to phosphorylate tau protein both *in vitro* and *in vivo* (Ferrer et al., 2005). Therefore, inhibition of GSK-3β alone is insufficient to inhibit abnormal tau hyperphosphorylation (Wang J. Z. et al., 2007). Less specific GSK-3β inhibitors that can also inhibit other tau kinases in addition to GSK-3β could lead to significant inhibition of abnormal tau hyperphosphorylation. An example of multi-target natural compounds is GV-971, which is a natural oliogomannate isolated from marine algae and has been approved by the Chinese FDA for the treatment of AD. This compound has been demonstrated to de-aggregate Aβ (Hu et al., 2004), to prevent astrocyte-mediated inflammatory responses (Wang S. et al., 2007), and to modulate intestinal bacteria (Wang et al., 2019), all of which have been reported to be involved in the pathogenesis of AD.

Attempts have been made to design and synthesize compounds that contain two distinct functional groups that each target a different pathway involved in AD (Cabrera-Pardo et al., 2019; Bhatia et al., 2021). This can be achieved by the molecular hybridization of different pharmacophore moieties from already identified bioactive molecules (Uddin et al., 2021). Each pharmacophore of the new hybrid drug can preserve the capacity of interacting with their specific sites on the targets and thus generate multiple specific pharmacological responses. This approach can eliminate the need to simultaneously administer multiple drugs with potentially different degrees of bioavailability, pharmacokinetics, and metabolism. Thus, this pharmacological approach can also provide patients with a simplification of the therapeutic regimen. Active immunotherapy against both amyloid pathology and tau pathology in a single bivalent AD vaccine is worth investigating.

Second, screen natural substances with multiple actions against various insults/mechanisms involved in AD. Several natural compounds, such as chelerythrine (Marasco et al., 2021), chalcone (Thapa et al., 2021), coumarin (Li et al., 2022), huprine (Serrano et al., 2016), curcumin (Mukherjee et al., 2021), rhein (Li et al., 2019), berberine (Akbar et al., 2021), and resveratrol derivatives (Akbar et al., 2021), have shown such potential and warrant further studies for AD drug development (Noori et al., 2021). The new drug GV-971 (Xiao et al., 2021) belongs to this category.

Third, develop a combination “cocktail” therapy with multiple drugs that target distinct mechanistic pathways of AD. This approach for the multi-target drug development strategy for AD is the use of cocktail therapy in AD clinical trials. Although single-target drugs, if used singly, may not be effective against AD because they inhibit only one of several disease pathways involved in an individual AD case, simultaneous treatments with more than one drug targeting distinct insults/mechanisms might have significant efficacy, according to the multifactorial AD hypothesis (Figure 1). Such an approach had been used effectively in chemotherapy for cancers and in fighting against HIV/AIDS as the cocktail therapy. It is well justified to employ such an approach for combating against AD, because of its multiple etiologies and disease mechanisms. Several clinical trials of combination therapy for AD have been carried out or are in process. Most of them comprise the concomitant use of memantine and a cholinesterase inhibitor (Kabir et al., 2020).

Fourth, employ a molecule that regulates multiple signaling pathways. There are molecules or factors that can modulate several molecular pathways associated with neurodegeneration and progression of AD. One example is brain insulin signaling, which has been shown to regulate neurodevelopment, neural plasticity, neuronal survival, and neurodegeneration (Chen et al., 2014). Brain insulin signaling is dysregulated in AD brain (Steen et al., 2005; Liu et al., 2011), and this dysregulation appears to contribute to impaired brain glucose metabolism (Chen et al., 2014), a well-established metabolic abnormality that precedes AD and is likely involved in neurodegeneration in AD. Restoration of brain insulin signaling by means of intranasal insulin treatment or of oral drugs that promote insulin sensitivity are under active investigation for the treatment of AD (Chen et al., 2016). Another example of this approach is to restore brain protein O-GlcNAcylation, a modification of proteins with β-N-acetyl glucosamine (GlcNAc) at the hydroxyl groups of serine or threonine residues of proteins (Butkinaree et al., 2010). O-GlcNAcylation modulates several pathways involved in AD pathogenesis, such as APP processing (Jacobsen and Iverfeldt, 2011), tau phosphorylation (Liu et al., 2004, 2009), synaptic integrity (Tallent et al., 2009), and insulin signaling (Yang et al., 2008). O-GlcNAcylation of both tau and global proteins is decreased in AD brain (Liu et al., 2004, 2009). Restoration of brain O-GlcNAcylation has been shown to improve cognitive function of mouse models of AD (Yuzwa et al., 2014; Pan et al., 2021). Finally, neurotrophic agents that can promote neurogenesis through multiple pathways have huge potential for treating AD. A neurotrophic peptidergic compound, P021, which could rescue neurogenesis and neuronal plasticity deficits, was found to reverse cognitive impairment, prevent amyloid plaque and tau pathologies, and dramatically reduce the rate of mortality in 3xTg-AD, a transgenic mouse model of AD (Kazim et al., 2014; Baazaoui and Iqbal, 2017).

## Approaches for Improving Multi-Target Strategies for Treating Alzheimer’s Disease

Extensive research during the last three decades has provided many insights into the possible molecular mechanisms underlying AD, although a complete understanding of the disease mechanisms is still far from being achieved. Nevertheless, brain imaging, biomarkers, and bioinformatics have provided useful information that can certainly help improve the efficacies of the multi-target strategy for treating AD.

The multifactorial nature of AD dictates the heterogenicity of the molecular mechanisms, pathologies, and clinical symptoms of AD patients. It is therefore possible to stratify AD cases into various subgroups that represent different etiologies, mechanisms, and clinical signs. By analyzing the CSF biomarkers, including the levels of Aβ, tau, and ubiquitin, we were able to stratify AD cases into five subgroups (Iqbal et al., 2005), each of which presents a different clinical profile, suggesting that the disease mechanisms among these groups might not be the same. Indeed, a recent tau immunotherapy clinical trial found effective mostly in those patients who had increased CSF tau levels (Novak et al., 2021). Intranasal insulin also showed different efficacies in ApoE4 carriers as compared to non-carriers (Reger et al., 2006). Very significant advances have been made during the last decade in the studies of AD brain imaging, CSF and plasma biomarkers, and genetic risk factors. These achievements can and should be used for stratifying patients for future AD clinical trials.

Repurposing of drugs that have been approved by the FDA for other conditions is currently being actively investigated for the treatment of AD. Several groups of drugs, such as anti-inflammatory drugs and anti-diabetic drugs, are currently under investigation for their potential efficacies for treating AD (Norins, 2021). A rationale for this approach is that the pathways the drugs target are involved in the mechanisms of AD. Especially if the so-called off-target effects of these drugs are also involved in neurodegeneration, then they have bigger potential for treating AD because they have multi-target properties. The repurposing approach is not only very attractive but also is supported by many investigators who are searching for an unconventional approach to AD drug development as well as by the NIH. In early November 2021, the National Institute on Aging organized an investigators’ meeting entitled Translational Bioinformatic Approaches to Drug Repurposing and Combination Therapy Development for AD/ADRD, where many promising advances were presented. Although previous clinical trials in which drug candidates were employed individually have failed to show significant efficacies against AD, retrospective studies using network-based approaches to large cohorts of human data indicate significant benefits of these drugs in preventing, and probably also treating, AD and dementia (Paranjpe et al., 2019). The discrepancy between the negative human trials with individual drugs and the positive retrospective human studies may again suggest the need for combinational treatments.

Precision medicine is a concept for treating individual patients according to their specific genetic, epigenetic, metabolic, environmental, and social profiles. Completion of human genome studies and the reasonable costs for individual genomic screening, together with recent advances in brain imaging and various biomarkers, make it feasible to employ the precision medicine approach for treating AD. This approach is uniquely helpful in the use of multi-target and cocktail therapy because the targets can be designed on the basis of the genetic, epigenetic, and metabolic information of the individual patients.

## Perspectives

Alzheimer’s disease drug development has reached a critical stage when critical reviews of previous efforts in combination with breakthrough thinking are required. Considering that aging is the most important risk factor for AD and that physical exercise is among the most effective prevention measures against the disease, both of which involve multiple mechanisms and pathways, the multi-target strategy for future AD drug development is clearly warranted. Simply shifting the target from amyloid β to tau or to any other single target might not succeed. Recent efforts in drug repurposing and combination therapy development for AD through analyzing big data of electronic health records in combination with genetic-based and omic-based approaches may be more likely to lead to the discovery of effective AD drugs.

Limited recent studies have already provided promising evidence for the multi-target strategy. A daily, fixed-dose combination of memantine extended-release (an N-methyl-D-aspartate receptor antagonist) and donepezil (a cholinesterase inhibitor) has shown some advantage. This combination, named Namzaric™, was approved by the FDA in 2014 for treating patients with moderate-to-severe AD (Deardorff and Grossberg, 2016).

The transition to a multi-target strategy for AD drug development faces many challenges, like any other paradigm shift does. Compared to clinical trials for single-target AD drug candidates, clinical trials for multi-target drug candidates and combination therapy are more complex and more expensive, require different designs, and produce data that are more difficult to interpret. Finally, the FDA may not readily accept drug approval applications that do not offer a clear single target or clear mechanism. However, in light of the desperate demand to fight such a devastating disease of multifactorial nature, there may be no better approach than the unconventional multi-target strategy for AD drug development.

## Author Contributions

C-XG drafted the manuscript. C-LD, FL, and KI reviewed, discussed, and performed the critical editing. All authors read and approved the final manuscript.

## Conflict of Interest

The authors declare that the research was conducted in the absence of any commercial or financial relationships that could be construed as a potential conflict of interest.

## Publisher’s Note

All claims expressed in this article are solely those of the authors and do not necessarily represent those of their affiliated organizations, or those of the publisher, the editors and the reviewers. Any product that may be evaluated in this article, or claim that may be made by its manufacturer, is not guaranteed or endorsed by the publisher.
